# New Journal

**Published:** 1874-02

**Authors:** 


NEW JOURNAL.—The Vermont Medical *Journal*, edited and published by J. M. Currier, M. D., Burlington, Vt., is a new candidate for professional support. We wish it success.

